# Self-efficacy as a mediator between dementia knowledge and screening intention among American Indian adults

**DOI:** 10.1093/geroni/igaf131

**Published:** 2025-12-04

**Authors:** Heehyul E Moon, Yeon-Shim Lee, Soonhee Roh, Cole Allick, James E Galvin, Sasheen T Stone

**Affiliations:** Kent School of Social Work and Family Science, University of Louisville, Louisville, Kentucky, United States; School of Social Work, San Francisco State University, San Francisco, California, United States; Department of Social Work, University of South Dakota-Sioux Falls, Sioux Falls, South Dakota, United States; College of Veterinary Medicine, Washington State University, Everett, Washington, United States; Department of Neurology, Comprehensive Center for Brain Health, Miller School of Medicine, University of Miami, Boca Raton, Florida, United States; Yankton Sioux Tribe, Wagner, South Dakota, United States

**Keywords:** ADRD screening intention, American Indian communities, Median analysis

## Abstract

**Background and Objectives:**

While early detection of Alzheimer’s disease and related dementias (ADRD) can help delay progression and improve outcomes, limited research is available on dementia-related health behavior, such as screening intention among American Indian/Alaska Native communities. Guided by the Theory of Reasoned Action, the Theory of Planned Behavior, and the Health Belief Model, this study examines whether self-efficacy mediates the association between dementia knowledge and intention to seek ADRD screening among American Indian adults.

**Research Design and Methods:**

Using a community-based participatory research approach, a cross-sectional survey was conducted with 248 American Indian adults (18 years and over) from a partner tribal community in the Northern Plains region in 2024. Measures included dementia knowledge, self-efficacy, screening intention, perceived susceptibility, stigma, and demographic factors. Mediation was tested using the Baron and Kenny framework and Sobel-Goodman tests.

**Results:**

Dementia knowledge significantly predicted both ADRD screening intention and self-efficacy. Self-efficacy also significantly predicted screening intention and partially mediated the relationship between knowledge and intention. Approximately 32% of the effect of dementia knowledge on screening intention was mediated by self-efficacy.

**Discussion and Implications:**

Findings underscore self-efficacy as a critical mechanism through which dementia knowledge translates into ADRD screening intention in American Indian communities. Interventions to promote early ADRD detection could enhance both knowledge and individual confidence. Future research should include more diverse groups within American Indian communities to identify common and unique dynamics among knowledge, self-efficacy, and screening intention, informing effective intervention strategies to reduce stigma and confidence in seeking timely ADRD screening.

Innovation and Translational Significance: This study addresses a significant gap in dementia prevention by identifying self-efficacy as a key mediating mechanism between dementia knowledge and screening intention for Alzheimer’s disease and related dementias among American Indian adults. Guided by established behavioral theories and implemented through a community-based participatory research framework, the study provides theoretically grounded and culturally informed insights into modifiable factors that can enhance early detection efforts. The findings offer practical strategies for developing targeted, community-led interventions aimed at increasing screening engagement among underserved populations.

Approximately 6.9 million Americans are projected to be living with Alzheimer’s disease and related dementias (ADRD) in 2025, the most common cause of dementia (Alzheimer’s Association, 2025). As the population ages, the number of adults aged 65 and older is expected to grow by 47% from 2022 to 2050, and those aged 85 and older by 84%, further driving the rise in ADRD cases (Alzheimer’s Association, 2025; Galvin, 2018). This surge has significant implications for individuals, families, and the healthcare system. The USC Schaeffer Center (2025) estimates that total dementia-related costs will reach $781 billion (in U.S. dollars), including $232 billion in healthcare expenses and $233 billion in unpaid caregiving. Lost earnings among caregivers total $8 billion, with quality-of-life losses adding $308 billion in societal costs. Thus, ADRD is a growing clinical and public health challenge and a major economic and societal burden, underscoring the urgent need for early detection, improved care strategies, and culturally responsive interventions, particularly for underserved populations.

Early detection and diagnosis can mitigate these impacts (Galvin et al., 2020; Lee et al., 2022; Park et al., 2020). Identifying ADRD early allows individuals, care partners, and family to take action to support brain health, engage in shared decision-making about their care, and access and utilize appropriate medical and social resources. Timely diagnosis can lead to delayed progression, improve outcomes, and help individuals remain in the community longer.

## Alzheimer’s disease and related dementias in American Indian/Alaska Native populations

While ADRD affects all communities, its impact on American Indian and Alaska Native populations represents a serious public health concern. Recent national estimates indicate that approximately one in six Native American adults aged 45 and older report experiencing memory or cognitive difficulties that may indicate early signs of dementia (Alzheimer’s Association, 2022). As life expectancy increases, the number of American Indian/Alaska Native individuals aged 65 and older living with dementia is projected to quadruple by 2060, drawing critical attention to the need for timely detection, diagnosis, and culturally responsive care within these communities. Despite the heterogeneity of American Indian/Alaska Native populations, many experience disproportionately high rates of ADRD risk factors, including hypertension, type 2 diabetes, high cholesterol, obesity, and tobacco use (Anstey et al., 2011; Garrett et al., 2015; Marden et al., 2017; Rönnemaa et al., 2011). These clinical risks, compounded by social determinants of health and structural inequities, contribute to the persistent underdiagnosis and underreporting of ADRD among American Indian/Alaska Native populations.

## Health services for American Indian/Alaska Native communities and ADRD screening behavior

Over 570 federally recognized tribes exist in the United States, each with distinct cultural, social, and healthcare contexts that shape access to and perceptions of ADRD screening. The Indian Health Service (IHS), a federal program fulfilling treaty obligations, provides healthcare to American Indian/Alaska Native communities through IHS-operated facilities, tribally managed programs, and urban Indian health centers. However, chronic underfunding, dependence on external referrals for specialty care, and systemic underinvestment have created significant service gaps, including inadequate ADRD screening capacity. These disparities are compounded by multiple barriers, including limited trust in healthcare systems, the absence of culturally appropriate diagnostic tools, and low awareness of ADRD symptoms among both patients and providers. American Indian/Alaska Native populations experience higher rates of misdiagnosis, while stigma, cultural misconceptions, and poor access to care delay diagnosis and treatment (Alzheimer’s Association, 2021). Collectively, these challenges highlight the critical need for research on self-efficacy and culturally grounded interventions to improve early ADRD detection in American Indian/Alaska Native communities.

## ADRD knowledge and screening behavior

Research shows that greater knowledge of ADRD is associated with a stronger intention to pursue screening. Park et al. (2020) found that individuals with higher ADRD knowledge were more likely to express willingness to be screened, suggesting that knowledge influences early detection behaviors. Galvin et al. (2007) similarly reported that awareness of memory changes was associated with engagement with brief cognitive screening tools such as the Eight-item Interview to Differentiate Aging and Dementia (AD8). These findings suggest that increasing ADRD knowledge is a key lever for enhancing early detection efforts. However, many studies focus on non-Hispanic White participants, limiting generalizability. American Indian/Alaska Native communities are underrepresented in national datasets, and epidemiological data on ADRD prevalence are scarce due to underreporting (Conniff et al., 2024; Johnson et al., 2023; Kirkpatrick et al., 2019). Public awareness campaigns often fail to change long-term behavior, and reliable early detection tools accessible across diverse populations remain limited (Apostolou et al., 2024; Friedman et al., 2009; Jernigan et al., 2020). For example, culturally relevant tools for assessing cognitive decline, such as the Community Screening Interview for Dementia (Hall et al., 2000), are rarely validated for use among the American Indian/Alaska Native populations. Combined with limited tribal engagement in study design, these factors hinder the development of long-term, contextually grounded research (Allick & Bogic, 2024; Racine et al., 2021).

Nevertheless, existing research points out that ADRD-related health behavior and knowledge in Indigenous populations are shaped by cultural beliefs and practices (Johnston et al., 2020). Within American Indian/Alaska Native communities, studies show low awareness of ADRD (Allick, 2024; Boyd et al., 2022; Laditka et al., 2009; Verney et al., 2008). Although this population is at elevated risk for dementia due to comorbidities such as diabetes and cardiovascular disease (Carty et al., 2020; Kirkpatrick et al., 2019; Nianogo et al., 2022; Olson & Albensi, 2020; Panegyres & Chen, 2014), care-seeking behavior may be hindered by historical mistrust, geographic isolation, or limited awareness (Allick, 2024; Crouch et al., 2023; Johnson et al., 2023).

## Self-efficacy and screening behavior

Self-efficacy is the belief in one’s capacity to perform the behaviors necessary to achieve specific outcomes (Bandura, 1977, 2006). It shapes how individuals think, feel, and act in health contexts. Individuals with higher self-efficacy are more likely to engage in self-care behaviors, adhere to medication and physical activity routines, and participate in screening programs (Aljasem et al., 2001; Lev, 1997). When individuals believe they can successfully perform a health-related behavior, they are more likely to initiate and maintain that behavior.

Research demonstrates that self-efficacy is a significant predictor of screening and disease management behaviors, including in the context of dementia. For instance, Park et al. (2020) found that higher self-efficacy significantly predicted intention to be screened for Alzheimer’s disease among nondemented older adults, even when accounting for attitudes, perceived norms, and control beliefs. Their study, based on the Integrated Behavioral Model, identified self-efficacy as a key mediating factor between psychosocial determinants and screening intention. Similarly, Galvin et al. (2007) reported that individuals who felt confident in recognizing memory changes were more likely to engage with dementia screening tools. These findings suggest that individuals who believe they can manage the screening and follow-up care process are more likely to pursue cognitive evaluation. Self-efficacy thus not only influences whether someone intends to undergo dementia screening, but also their ability to follow through, especially when facing emotional, structural, or cultural barriers.

Despite the recognized importance of self-efficacy in health behavior, research gaps remain. American Indian/Alaska Native populations are often underrepresented in health behavior research, resulting in limited data on effective interventions for these communities (Conniff et al., 2024; Moy et al., 2006). Although culturally tailored interventions exhibit promise, additional research is needed on how to effectively adapt health promotion programs to American Indian/Alaska Native cultures (Lewis et al., 2021; Nahian & Jouk, 2025; Webkamigad et al., 2020). Tools to measure self-efficacy and health literacy are rarely adapted to reflect values, worldviews, and languages of the American Indian/Alaska Native populations (Lewis et al., 2021). More research on how traditional conceptualizations can be integrated with conventional healthcare to enhance self-efficacy and dementia-related health outcomes is needed (Webkamigad et al., 2020).

## Theoretical frameworks: Theory of Reasoned Action and Theory of Planned Behavior, and Health Belief Model

The Theory of Reasoned Action (TRA) and Theory of Planned Behavior (TPB) provide a foundational framework for understanding intention to engage in health-related behaviors, including dementia screening. TRA posits that behavior is driven by behavioral intentions, which are shaped by attitudes and perceived social norms (Adams et al., 2022; Galvin et al., 2007, 2010; Montano & Kasprzyk, 2002). TPB expands this by adding perceived behavioral control, which accounts for an individual’s confidence in their ability to perform the behavior (Adams et al., 2022; Galvin et al., 2007, 2010; Montano & Kasprzyk, 2002). These frameworks are especially relevant for dementia screening in American Indian/Alaska Native communities, where attitudes, community norms, and perceived control may strongly influence willingness to pursue an official diagnosis. Screening intention, therefore, serves as a meaningful proxy for eventual screening behavior in this context.

The Health Belief Model (HBM) is another widely utilized theoretical framework in health research. It posits that an individual’s likelihood of engaging in preventive health behaviors, such as dementia screening, is influenced by a combination of perceived susceptibility to and severity of illness, perceived benefits and barriers to action, perceived threat of the illness, and self-efficacy, or confidence in one’s ability to overcome barriers (Jung & Gu, 2024; Rosenstock et al., 1988). Perceived susceptibility refers to an individual’s belief about the likelihood of developing a particular disease or health condition. For instance, individuals who believe they are at high risk for developing dementia (high perceived susceptibility) and view the disease as serious or life-threatening (high perceived severity) are more likely to engage in early screening and diagnostic behaviors. Self-efficacy, added later to the original HBM, refers to the belief in one’s capability to take necessary health actions, such as adopting regular exercise routines. While limited research has applied the HBM specifically to American Indian populations in the context of dementia, related studies suggest the model’s applicability. For example, research with American Indian women found that greater motivation and fewer perceived barriers were associated with increased cervical cancer screening (Lee et al., 2020). Similarly, among African American women, a higher perception of screening benefits and fewer barriers were linked to increased colon cancer screening rates (Frank et al., 2004). These findings highlight the potential relevance of HBM in understanding preventive health behaviors for dementia across diverse populations.

Guided by TRA and TPB and the HBM, the current study tests the following hypotheses using the Baron and Kenny (1986) mediation framework:

**H1:** Greater dementia knowledge will be significantly associated with higher intention to seek ADRD screening.

**H2:** Greater dementia knowledge will be significantly associated with higher self-efficacy regarding ADRD screening.

**H3:** Higher self-efficacy will be significantly associated with greater intention to seek ADRD screening, controlling for dementia knowledge.

**H4:** Self-efficacy will mediate the relationship between dementia knowledge and intention to seek ADRD screening.

## Method

### Study design

A community-based participatory research (CBPR) approach was employed to guide the study design and implementation. The data used in the current analysis are part of a larger CBPR initiative developed in partnership with the partner tribal community in the Northern Plains region. A cross-sectional survey was conducted in September 2024 among American Indian adults (*N *= 248) living in the Northern Plains region. Participants were recruited using a combination of convenience and snowball sampling strategies through community-based avenues such as the tribal powwow, referrals, and word of mouth.

This study was conducted with the guidance of a Community Advisory Board (CAB), consisting of American Indian community elders and religious and community leaders. The CAB provided critical input on all aspects of the study, including survey development, implementation, recruitment strategies, and dissemination of findings to ensure cultural relevance and community priorities. The CAB met quarterly, and each member received $100 in compensation per meeting. Based on CAB recommendations, the minimum age for participation was lowered from 50 to 18 years.

Eligible participants were those who (a) self-identified as American Indian, (b) were 18 years of age or older, (c) were born and raised in, or currently resided on, the tribal community, and (d) demonstrated sufficient cognitive ability to understand and complete the survey, as determined using a validated 8-item AD8 screening tool administered during eligibility screening. The final sample completed a self-administered paper survey in locations chosen by participants for comfort and privacy. All data collected is considered joint property of the investigators and the Tribe. Institutional Review Board approval was obtained prior to data collection from the University of Louisville. The survey took approximately 20–30 min to complete, and all participants provided signed informed consent. A total of 248 adults from the partner tribal community participated in the study, and each received a $20 cash incentive for their participation.

### Measures

#### Intention to seek ADRD screening

Intention to be screened for ADRD was assessed using four items developed by Galvin et al. (2006, 2008). Sample items include: “I plan to have a screening test for memory loss at some point in my life,” “I plan to have a screening test for memory loss in the next year,” “I plan to have a screening test for memory loss after I reach a certain age,” and “I plan to have a screening test for memory loss if I experience symptoms.” Responses were recorded using a binary scale (0 = Disagree, 1 = Agree) with higher total scores indicating greater intention to seek screening. The scale demonstrated good internal consistency in this study (Cronbach’s α = 0.72).

#### Dementia knowledge

The 10-item Dementia Knowledge Subscale of the 20-item Dementia Attitudes Scale was used to measure dementia knowledge (DAS; O′Connor & McFadden, 2010). The DAS was originally developed to assess attitudes toward dementia for both college students and direct care workers (family and professional carers) and represents two major domains: Dementia knowledge (e.g., “People with ADRD can enjoy life.”) and social comfort (e.g., “I feel confident around people with ADRD.”). For the current study, response options were modified from a 7-point Likert scale ranging from 1 (strongly disagree) to 7 (strongly agree) to a binary option (0= disagree, 1 = agree). Summative scores were computed for the total, ranging from 0 to 10, indicating higher scores reflecting greater dementia knowledge. The scale shows high reliability (with Cronbach’s alpha coefficient ranging from 0.83 to 0.85) as well as good validity compared with other scales that measure attitude, making it a suitable research tool (O’Connor & McFadden, 2010). The Cronbach’s α of the scale was 0.84 in the present study.

#### Self-efficacy

Self-efficacy related to ADRD screening was assessed using a 7-item scale developed by Galvin et al. (2006). The scale measured participants’ perceived confidence in obtaining a screening test for memory loss. Items were rated using a binary response format (0 = Disagree, 1 = Agree), with higher scores indicating greater self-efficacy regarding ADRD screening. Example items include “I am confident I can get a screening test for memory loss if I have symptoms,” “I am confident I can ask my doctor for a referral for a memory screening test,” and “It is entirely my decision whether or not I get screened for memory loss.” In this study, the scale demonstrated good internal consistency (Cronbach’s α = 0.88).

#### Covariates

Participants reported their age using an open-ended item and identified their gender as either male or female. Marital status (0 = Never married, 1 = Married, 2 = Divorced, 3 = Widowed, 4 = Separated, 5 = Other), educational attainment (0 = Less than high school/GED, 1 = High school/GED, 2 = Some college/Associate degree, 3 = Bachelor’s degree, 4 = Master’s degree or higher), and monthly household income (seven categories ranging from 1 = Under $500 to 7 = $3,000 and over) were also collected and are described in the sample characteristics. Knowing someone with ADRD was assessed with the item, “Have you known someone with dementia?” (0 = No, 1 = Yes). Tribal tradition identity was measured with the item, “How much do you identify with your own tribal traditions?” (0 = Not at all, 1 = A little, 2 = Some, 3 = A lot). Religious/spiritual beliefs were measured with the item, “How important would you say your religious/spiritual beliefs are to you?” (0 = Not at all important, 1 = Somewhat important, 2 = Very important). Use of traditional Native remedies/practices was assessed with the item, “Do you use traditional Native remedies and/or practices to remain healthy and/or prevent illness?” (0 = No, 1 = Yes). These cultural and contextual variables were incorporated to provide a more comprehensive characterization of the sample. Although not part of the primary mediation model, the cultural variables (i.e., tribal tradition identity, religious/spiritual beliefs, and use of traditional Native remedies/practices) were examined descriptively and in supplementary exploratory analyses to explore their potential relationships with the study’s key constructs. Perceived susceptibility was assessed using four items adapted from prior work (Galvin et al., 2006). Participants rated their agreement with the following statements: (1) “Compared to other people my age, I have a pretty good chance of getting Alzheimer’s disease,” (2) “As I age, I am more likely to get alzheimer’s disease,” (3) “If a family member suffered from Alzheimer’s disease, then I will too,” and (4) “I feel the chances are good that I will get alzheimer’s disease.” Items were rated on a 5-point Likert scale ranging from 1 (strongly disagree) to 5 (strongly agree). Higher scores indicate greater perceived susceptibility to Alzheimer’s disease. A mean composite score was calculated for analysis, with higher scores reflecting stronger perceived risk. Ten items of Perceived Stigma and Social Impact were used to assess anticipated stigma and perceived social consequences of receiving an Alzheimer’s diagnosis ­(Galvin et al., 2006). Participants responded to hypothetical statements beginning with: “If you were suffering from Alzheimer’s disease…” Sample items include: “Would you rather people not know about your disease?” and “Would your neighbors or colleagues have less esteem for your family?” Responses were coded on a 3-point scale: 0 = No, 1 = Maybe, 2 = Yes. Items were reverse-coded as needed (e.g., support-related questions) so that higher scores consistently reflected greater perceived stigma or social impact. A total stigma score was computed by summing the item responses, with higher scores indicating greater perceived stigma and social consequences associated with ADRD.

### Analytic strategies

Our strategy is organized into two parts. First, descriptive statistics summarized the sample characteristics and key study variables (Table 1). Second, to test the hypotheses, we first determine whether self-efficacy mediates the relationship between dementia knowledge and intention to seek ADRD screening (Baron & Kenny, 1986). This approach involves establishing three conditions for mediation: (1) dementia knowledge must significantly predict the ADRD screening intention (pathway c, total effect); (2) dementia knowledge must significantly predict self-efficacy (pathway a); and (3) self-efficacy must significantly predict the ADRD screening intention while controlling for the dementia knowledge (pathway b). As the next step, the effect size of dementia knowledge on screening intention was assessed (pathway *c'*, direct effect). Then, A Sobel-Goodman test was subsequently used to assess the significance of the indirect effect. No multicollinearity problems were observed among the independent variables, as the variance inflation factor scores ranged from 1.04 to 1.24 (Mertler & Vannatta, 2002). This study used STATA 17 for data analysis. Correlation results among study variables are available in Supplementary Table 1 (see online supplementary material). Traditional and spiritual measures were not part of the primary mediation model but were examined in supplementary regression analyses to explore their associations with the outcome variable. Detailed results are provided in Supplementary Table 2 (see online supplementary material).

**Table 1.** Participant demographics and descriptive statistics for key study variables (*N *= 246).

Characteristics	Mean	SD	Min	Max	%
**Age (years)**	45	15	18	81	
**Gender (female)**					
**Marital status (married)**					
**Education (some college/associate degree/bachelor’s degree or higher)**					51
**Know someone with ADRD (Yes)**					25
**Born on a reservation**					83
**Tribal tradition identity (some/a lot)**					41
**Importance of religious/spiritual beliefs**					51
** Very important**					79
** Somewhat important**					
** Not at all**					57
**Use of traditional native remedies/practices (Yes)**					37
**Monthly household income**					6
** $3,000 and over**					56
**Dementia knowledge**	8.25	2.35	0	10	
**Self-efficacy to get screening**	4.83	1.88	0	6	36
**ADRD screening intention**	2.56	1.27	0	4	
**Perceived susceptibility**	3.32	1.92	0	6	
**Perceived stigma and social impact**	6.17	4.08	0	20	

*Note.* ADRD = Alzheimer’s disease and related dementias; *SD* = standard deviation.

## Results

### Descriptive statistics

The descriptive characteristics of the sample are presented in Table 1. The sample included 246 participants, with a mean age of 45 years (*SD *= 15, range = 18–81). Over half of the participants identified as female (51%), and 56% reported never having been married. Regarding education, 65% held a bachelor’s degree or less, with 8% having completed high school or a GED. More than two-thirds of the participants reported earning less than $3,000 per month (see Table 1). Over 40% of the respondents reported they had known someone with ADRD. Approximately half of the participants reported being born on a reservation, and 79% identified with tribal traditions to some or a great extent. 95% of the participants considered religious or spiritual beliefs very important or somewhat important, while 6% not important at all. In addition, 56% reported using traditional Native remedies or practices to remain healthy.

As for key study variables (Table 1), the mean score for dementia knowledge was 8.25 (*SD *= 2.35) out of a maximum of 10. Self-efficacy scores ranged from 0 to 6, with a mean of 4.83 (*SD *= 1.88). Perceived susceptibility to dementia ranged from 0 to 6 (*M *= 3.32, *SD *= 1.92), and perceived stigma ranged from 0 to 20 (*M * = 6.17, *SD *= 4.08). ADRD screening intention scores ranged from 0 to 4, with a mean of 2.56 (*SD *= 1.27).

To further explore participants’ screening intentions, item-level frequencies were examined. Most participants (73.4%) agreed with the statement “I plan to have a screening test for memory loss at some point in my life.” In contrast, fewer participants (37.1%) agreed that they planned to have a screening test within the next year. Screening intention was also high when symptoms were present (74.6%) or after reaching a certain age (64.1%).

### Mediation

**Figure 1.**
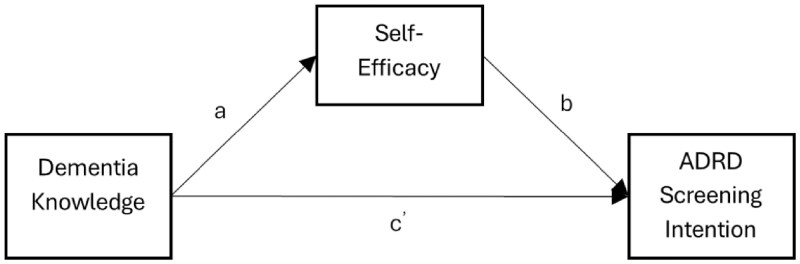
Mediation model of the relationship between dementia knowledge, self-efficacy, and ADRD screening intention. ADRD = Alzheimer’s disease and related dementias. This conceptual model illustrates self-efficacy as a mediator of the relationship between dementia knowledge and intention to seek ADRD screening. Arrows represent hypothesized directional pathways between variables.

To assess the mediating effect of self-efficacy on the associations between Dementia knowledge and intention to seek ADRD screening while controlling for age, gender, perceived susceptibility, perceived stigma, and social impact, a series of regression analyses was conducted following the Baron and Kenny approach (Figure 1). As hypothesized, linear regression (pathway c in Table 2) showed that dementia knowledge significantly predicted intention to seek ADRD screening (β  =  0.125, *SE* = .034, *p* < .001), indicating that individuals with higher dementia knowledge reported greater screening intention. In the second step (pathway a in Table 2), dementia knowledge was a significant predictor of self-efficacy (β  =  0.251, *SE* = .047, *p* < .001), suggesting that greater knowledge about dementia is associated with increased confidence in one’s ability to seek screening. In the third step, both dementia knowledge and self-efficacy were entered into the model predicting screening intention. Self-efficacy remained a significant predictor of screening intention (pathway b in Table 2; β  =  0.158, *SE* = .045, *p* = .001). The effect of dementia knowledge was reduced but still significant (pathway *c’* in Table 2; β  =  0.085, *SE* = .035, *p* = .014), indicating partial mediation.

**Table 2.** Mediation analysis: self-efficacy as a mediator of the relationship between dementia knowledge and ADRD screening intention (*N *= 246).

Pathway	Β	*SE*	*t*/*z*	*p*	95% CI
**Direct and mediated effects**					
**Dementia knowledge → Self-efficacy (*a*)**	.251	.047	5.35	<.001	[.158, .344]
**Self-efficacy → Screening intention (*b*)**	.158	.045	3.49	.001	[.069, .248]
**Dementia knowledge → Screening intention (*c'*)**	.085	.035	2.45	.014	[.017, .154]
**Indirect effect (*a* × *b*)**	.040	.014	2.92	.003	—
** Total effect (*c*)**	.125	.034	3.71	<.001	[.058, .192]
**Model statistics**					
***R*² (full model)**	.127	—	—	—	—
** Adjusted *R*²**	.105	—	—	—	—
***F* (6, 239)**	5.78	—	—	<.001	—
**Effect size ratios**					
**Proportion mediated**	31.8%	—	—	—	—
**Indirect/direct effect ratio**	0.465	—	—	—	—
**Total/direct effect ratio**	1.465	—	—	—	—

*Note*. All models control for age, gender, perceived susceptibility, perceived stigma, and social impact of ADRD. Perceived stigma was significantly and negatively associated with self-efficacy (*p* < .05). Perceived susceptibility was a significant positive predictor of screening intention in the total effect model (*p* = .02) and in the final model (*p* = .05). Age and gender were not significant predictors in any of the models. The indirect effect was tested using the Sobel-Goodman method. ADRD = Alzheimer’s disease and related dementias; β = standardized regression coefficient; CI = confidence interval; *SE* = standard error.

A Sobel-Goodman test confirmed the presence of a significant indirect effect of dementia knowledge on screening intention through self-efficacy (indirect effect = 0.040, *SE* = 0.014, *p* = .003). Approximately 31.8% of the total effect of dementia knowledge on screening intention was accounted for by self-efficacy. The ratio of the indirect to direct effect was 0.465, and the ratio of the total to direct effect was 1.465.

## Discussion

This study employed a community-based participatory research approach to enhance recruitment and develop culturally grounded survey instruments in collaboration with diverse Community Advisory Board members from the partner tribal community in the Northern Plains region, building on a 7-year research partnership. Recognizing the critical role of early ADRD screening in reducing individual, familial, and societal burden and in facilitating advanced care planning, the study examined factors influencing ADRD screening initiation among American Indian adults in the tribal community.

Consistent with prior research (Galvin et al., 2006, 2008; Park et al., 2020), this study identified self-efficacy, specifically, confidence in performing screening, as a significant partial mediator of the association between dementia knowledge and ADRD screening intention among American Indian adults in the tribal community. Although knowledge of ADRD causes, symptoms, and consequences remained a significant direct predictor after accounting for self-efficacy, more than 30% of its effects were explained indirectly, underscoring the critical role of confidence in seeking, accessing, and deciding to undergo memory loss screening. These findings also support Bandura’s self-efficacy theory, TRA/TBP, and HBM, highlighting confidence as a key mechanism linking knowledge to actionable behavior. While knowledge raises awareness of risks and benefits, self-efficacy may enable individuals to believe they can successfully navigate the healthcare system, control the process, make their screening decision, and manage the outcomes of screening, thus bridging the gap between awareness and intention among American Indian adults in the tribal community. Thus, culturally responsive interventions should address not only educational content but also individuals’ confidence in navigating the healthcare system, including IHS, and their beliefs in their ability to seek screening.

Perceived susceptibility to ADRD, as one of the covariates, was positively associated with screening intention. Consistent with HBM and previous research, heightened risk perception may encourage proactive action, such as early screening, as individuals seek to mitigate potential future impacts on cognitive health and quality of life. Although not included among the primary study variables, additional analyses revealed a positive correlation between perceived susceptibility and the total number of chronic conditions (*r* = .20, *p* < .001). This finding suggests that individuals with multiple health conditions may perceive a heightened risk for diseases such as ADRD and may consequently be more motivated to engage in preventive health behaviors, including screening. Thus, integrating memory screening tools and facilitating easy accessibility to ADRD screening and professional communications through routine health visits or community health events may create an optimal environment for these communities. Given the diversity of healthcare access points within the I/T/U system of IHS and variations in local healthcare resources, understanding the specific healthcare ecology of each Tribal community is critical for designing effective, culturally grounded ADRD screening interventions. These findings carry significant implications for practice. Community leaders, tribal health organizations, and service providers can leverage these insights to advance dementia education and early detection efforts. Dementia education could be integrated into existing health programming, such as chronic disease management initiatives or elder wellness programs. Community health workers, respected Elders, or peer educators could receive training to deliver culturally grounded information that enhances self-efficacy and reduces stigma surrounding care-seeking behaviors. Efforts to increase screening uptake should extend beyond awareness-raising to prioritize strengthening individual self-efficacy—specifically, confidence in recognizing symptoms, navigating healthcare systems, and making informed decisions about memory screening. Community-based initiatives that normalize discourse around memory loss and support agency in health decision-making may prove particularly effective within Indigenous contexts, where collective values and relational approaches to health are central.

Perceived stigma and social impact, as covariates, were not significantly associated with screening intention but with self-efficacy. The stigma scale used in this study assessed multiple dimensions of anticipated social and emotional reactions to a potential Alzheimer’s disease diagnosis, including concerns about disclosure, anticipated shame or embarrassment, fears of social rejection, and perceptions of diminished family support. These items captured both internalized stigma and anticipated public stigma. Potentially consistent with Social Cognitive Theory (Bandura, 1986), environmental and social factors such as anticipated rejection or judgment can diminish individuals’ beliefs in their ability to take action, particularly for sensitive health behaviors like cognitive screening. When individuals expect stigma from family, friends, or the community, they may experience reduced agency, leading to lower self-efficacy in navigating healthcare systems, making decisions, and seeking care. Future research should explore how different dimensions of stigma related to dementia (e.g., internalized stigma, culturally specific beliefs) influence both self-efficacy and preventive health behaviors related to cognitive health and how these are associated with family dynamics, cultural norms, and trust in health care systems across different tribes to inform the effective intervention strategies to reduce stigma and confidence in seeking timely ADRD screening.

Age and gender were not significant predictors in any of the models. The findings suggest that psychological and contextual factors, such as perceived risk, stigma, and self-efficacy, may play a more central role in shaping ADRD screening intentions than demographic characteristics. Supplementary analyses incorporating cultural and spiritual measures suggested that engagement in traditional Native remedies and practices may be positively associated with ADRD screening intention (*p* = .061). Although exploratory, this trend highlights the potential value of integrating traditional healing and cultural knowledge into dementia education and screening initiatives. Detailed results are presented in Supplementary Table 2 (see online supplementary material).

This study has several limitations. First, the cross-sectional design limits causal interpretations of the observed relationships between dementia knowledge, self-efficacy, stigma, and screening intentions. Longitudinal studies are needed to establish temporal sequencing and causal pathways. Second, the study was conducted with participants from a single tribal community, which may limit the generalizability of findings to other American Indian or Alaska Native groups with different cultural, social, and healthcare contexts. Third, while the sample included a wide age range, cultural perceptions of dementia may vary across generations, potentially influencing screening attitudes in ways not fully captured in the current analysis. Finally, while variables including tribal identity, traditional medicine use, and income were collected and described to characterize the sample, they were not incorporated into the multivariable mediation model. These factors may influence dementia knowledge, self-efficacy, and screening intentions in complex ways. Future studies employing larger and more diverse samples should examine how these contextual and cultural characteristics interact with psychological constructs to shape ADRD screening behaviors in Indigenous communities. Future research should expand beyond a single tribal community to examine how dementia knowledge, perceived stigma, and self-efficacy may vary across diverse American Indian and Alaska Native populations over time. While historical experiences such as colonization, historical trauma, and structural barriers to healthcare may create commonalities in health beliefs and behaviors, important cultural, linguistic, and social differences exist among tribal nations. These differences likely shape perceptions of dementia, willingness to engage in screening, and preferred approaches to care planning. In addition, qualitative research exploring topics such as how individuals define dementia or how they would prefer to approach a dementia diagnosis could provide deeper insight into culturally specific meanings, values, and experiences related to cognitive health (Jacklin & Walker, 2020).

## Conclusion

Given the rapid increase in the aging American Indian and Alaska Native population and the growing burden of ADRD, this CBPR-based study provides critical insights into screening intentions within a historically underserved group. Findings highlight self-efficacy as a key mechanism linking dementia knowledge to screening intention, while perceived risk and stigma may shape behavioral motivation. Future efforts should prioritize culturally responsive interventions that strengthen self-efficacy, reduce stigma, and reflect the diverse cultural understandings of dementia across tribal communities.

## Supplementary Material

igaf131_Supplementary_Data

## Data Availability

The data are not publicly available due to the community-based nature of the research, data governance agreements with partnering tribes, and ethical restrictions related to participant confidentiality and Institutional Review Board approval. The current was not preregistered.
