# Supplementary material for: Self-efficacy as a mediator between dementia knowledge and screening intention among American Indian adults
Source: Innov Aging. 2025 Dec 4;10(1):igaf131. doi: 10.1093/geroni/igaf131 (PMC12782657; doi:10.1093/geroni/igaf131)
Supplement: igaf131_Supplementary_Data [file igaf131_supplementary_data.docx]

***Innovation in Aging* Supplementary Material: Moon, Lee, Roh, Allick, Galvin, & Stone.**

**Self-Efficacy as a Mediator Between Dementia Knowledge and Screening Intention Among American Indian Adults.**

**Supplementary Table 1. Correlation among Study Variables (*N* = 246)**

| **Variable** | **1** | **2** | **3** | **4** | **5** | **6** | **7** |
| --- | --- | --- | --- | --- | --- | --- | --- |
| 1. ADRD Screening Intention | — |  |  |  |  |  |  |
| 2. Dementia Knowledge | .24*** | — |  |  |  |  |  |
| 3. Self-Efficacy | .30*** | .35*** | — |  |  |  |  |
| 4. Gender | –.01 | .05 | .12 | — |  |  |  |
| 5. Age | .06 | .13* | .16* | .01 | — |  |  |
| 6. Perceived Susceptibility | .15* | .08 | .19** | .17** | .30*** | — |  |
| 7. Perceived Stigma & Social Impact | –.06 | –.09 | –.23*** | –.02 | –.13* | –.09 | — |

Note. ADRD = Alzheimer’s disease and related dementias.

****p* < .001; ***p* <.01; **p* < .05

**Supplementary Table 2. Regression Predicting ADRD Screening Intention Including Cultural and Spiritual Measures (*n* = 245)**

| **Predictor** | **β** | **SE** | ***p*** | **95 % CI** |
| --- | --- | --- | --- | --- |
| Dementia Knowledge | 0.07 | 0.04 | .056 | [–0.00, 0.14] |
| Self-Efficacy | 0.15 | 0.05 | .001 | [0.07, 0.24] |
| Age | –0.10 | 0.16 | .532 | [–0.42, 0.22] |
| Gender | –0.14 | 0.16 | .374 | [–0.44, 0.17] |
| Perceived Susceptibility | 0.07 | 0.04 | .114 | [–0.02, 0.15] |
| Perceived Stigma | 0.01 | 0.02 | .691 | [–0.03, 0.05] |
| Tribal Tradition Identity | –0.00 | 0.01 | .849 | [–0.03, 0.02] |
| Use of Traditional Native Remedies/Practices (Yes) | 0.37 | 0.20 | .061 | [–0.02, 0.76] |
| Importance of Religious/Spiritual Beliefs | 0.18 | 0.13 | .174 | [–0.08, 0.44] |
| Constant | 0.64 | 0.42 | .133 | [–0.20, 1.48] |

Note. ADRD = Alzheimer’s disease and related dementias; β = unstandardized regression coefficient; SE = standard error; CI = confidence interval. Model statistics. *F*(9, 235) = 4.67, *p* < .001; R² = .15; Adjusted R² = .12; Use of traditional Native remedies/practices showed a positive trend (*p* = .061), suggesting that engagement in traditional healing may enhance motivation and confidence for ADRD screening. Tribal tradition identity and religious/spiritual beliefs were not statistically significant predictors.
